# Sympathetic Denervation Accelerates Wound Contraction but Inhibits Reepithelialization and Pericyte Proliferation in Diabetic Mice

**DOI:** 10.1155/2017/7614685

**Published:** 2017-09-24

**Authors:** Zhifang Zheng, Yu Wan, Yishu Liu, Yu Yang, Jianbing Tang, Wenhua Huang, Biao Cheng

**Affiliations:** ^1^The Graduate School of Southern Medical University, Guangzhou, China; ^2^Department of Plastic Surgery, Guangzhou General Hospital of Guangzhou Military Command, Guangzhou, China; ^3^Department of Anatomy, School of Basic Medicine Sciences, Southern Medical University, Guangzhou, China; ^4^The Graduate School of Third Military Medical University, Chongqing, China; ^5^Center of Wound Treatment, Guangzhou General Hospital of Guangzhou Military Command, Guangzhou, China; ^6^The Key Laboratory of Trauma Treatment & Tissue Repair of Tropical Area, PLA, Guangzhou, China

## Abstract

Previous studies focused on the effects of sympathetic denervation with 6-hydroxydopamine (6-OHDA) on nondiabetic wounds, but the effects of 6-OHDA on diabetic wounds have not been previously reported. In this study, treated mice received intraperitoneal 6-OHDA, and control mice received intraperitoneal injections of normal saline. Full-thickness wounds were established on the backs of mice. The wounds were sectioned (four mice per group) for analysis at 2, 5, 7, 10, 14, 17, and 21 days after injury. The wound areas in the control group were larger than those in the treatment group. Histological scores for epidermal and dermal regeneration were reduced in the 6-OHDA-treated group on day 21. The mast cells (MCs) in each field decreased after sympathectomy on days 17 and 21. The expression levels of norepinephrine, epidermal growth factor (EGF), interleukin-1 beta, NG2 proteoglycan, and desmin in the treatment group were less than those in the control group. In conclusion, 6-OHDA delays reepithelialization during wound healing in diabetic mice by decreasing EGF, but increases wound contraction by reducing IL-1*β* levels and the number of MCs. Besides, 6-OHDA led to reduced pericyte proliferation in diabetic wounds, which might explain the vascular dysfunction after sympathetic nerve loss in diabetic wounds.

## 1. Introduction

Diabetic foot ulcers (DFUs) are a common cause of hospitalization in diabetic patients [1]. Approximately 10–15% of diabetic patients develop foot ulcers, and 15% of DFU patients require amputation [2]. In contrast to nondiabetic wounds, diabetic wounds are characterized by prolonged inflammation, delayed wound closure [3], and impaired angiogenesis [4]. The neuroimmune axis and successful angiogenesis are essential for DFU healing [5, 6]. Although great progress has been made in the treatment of DFUs, they remain difficult to fully cure. Thus, there is a continuing need to understand the mechanisms behind DFUs and to explore new treatments.

Sympathetic efferents are important for the healing of DFUs. Sympathetic nerve damage is an important feature of diabetic neuropathy [7] and impairs vasomotor control and increases skin capillary permeability in diabetic patients [8]. Lumbar sympathectomy is a revascularization technique used in diabetic patients with ischemic feet [9]. However, the findings of Kokobelian et al. suggest that sympathectomy is ineffective for DFUs [10]. Therefore, the role of sympathetic nerve failure in the wound healing of DFUs remains controversial.

6-Hydroxydopamine (6-OHDA) is a specific sympathetic neurotoxin that selectively destroys peripheral sympathetic nerve termini. Nerve regeneration after chemical sympathectomy with 6-OHDA requires at least 1 month [11], which allows for continued wound observation after sympathetic denervation. Previous studies have shown the effects of 6-OHDA on nondiabetic wounds. Kim et al. [12] reported that 6-OHDA reduces inflammation and significantly delays epidermal wound healing in linear skin incisions in rats. Saburo et al. [13] suggested that 6-OHDA may decrease collagen metabolism and reduce the number of capillaries during the wound healing of animal burns. Souza et al. [14] found that intraperitoneal administration of 6-OHDA in 1% ascorbic acid accelerated wound contraction during cutaneous wound healing in rats. However, the effects of 6-OHDA on diabetic wounds have not been reported.

The wound-healing process includes an inflammatory change and proliferation phase, which includes fibroplasia, angiogenesis, and reepithelialization [15]. Angiogenesis during wound healing depends upon dynamic interactions between endothelial cells, angiogenic cytokines, and the extracellular matrix (ECM) [16]. NG2proteoglycan and desmin are pericyte markers in mice [17]. To explore the role of sympathetic denervation in the healing of DFUs, wound reepithelialization, wound contraction, collagen fibers, mast cell (MC) distribution, and the protein levels of norepinephrine (NE), epidermal growth factor (EGF), interleukin-1 beta (IL-1*β*), NG2 proteoglycan, desmin, and MMP-9 were observed in cutaneous wound healing after chemical sympathectomy with 6-OHDA in diabetic (db/db) mice.

## 2. Materials and Methods

### 2.1. Animals and Materials

Spontaneously diabetic female mice (*n* = 56, BKS.Cg-Dock7m^+/+^Leprdb/JNju, aged 6 weeks) were purchased from Nanjing Biomedical Institute, Nanjing University, Nanjing, China. Their diabetes was generated by a homozygous mutation in the leptin receptor gene, producing recognizable phenotypes characteristic of obesity and diabetes. The diabetic mice were maintained on a normal-fat and normal-sugar diet, and their blood sugar levels increased from 4 to 8 weeks of age. The fasting random glucose levels from the tail vein in the diabetic mice were measured in both 6-OHDA-treated and control groups during the experimental phase. 6-OHDA and ascorbic acid were purchased from Sigma-Aldrich (St. Louis, MO, USA). Primary antibodies for EGF, IL-1*β*, NG2 proteoglycan, desmin, and MMP-9were purchased from Abcam (Cambridge, UK), and a primary antibody for NE was purchased from Lifespan (Cambridge, UK).

### 2.2. Chemical Sympathectomy

The mice were housed for 1 week before use. The animals were maintained at 22–24°C under a 12/12 h light/dark cycle with free access to standard laboratory food and water. Animal experiments were conducted according to institutional guidelines and were approved by the animal care committee. The mice were divided randomly into 6-OHDA-treated and control groups. The treatment mice received intraperitoneal injections of 100 mg/kg 6-OHDA in 0.9% NaCl plus 10^−7^ M ascorbic acid on days −7 and −5 and 200 mg/kg 6-OHDA on day −3. Control mice received intraperitoneal injections of 0.9% NaCl plus 10^−7^ M ascorbic acid [11, 18].

### 2.3. In Vivo Wound Closure

The mice were anesthetized with 0.6% pentobarbital sodium (40 mg/kg), and skin punches were used to generate two rounds of full-thickness dermal wounds (0.6 cm in diameter, at a distance of at least 1.0 cm) on both sides of the dorsal trunk. After the wounding procedure, the animals were housed individually in separate cages. The wounds were imaged using a digital camera at 2, 5, 7, 10, 14, 17, and 21 days after injury. The wound sizes were measured using Image-Pro Plus software (ver. 6.0; Media Cybernetics Corp., Silver Springs, MD, USA) and calculated against the original area (on day 0), which was set at 100%. The wounds, including the tissue 2 mm around the edge of the wound, were sectioned under anesthesia (four mice per group) for analyses at each time point.

### 2.4. Hematoxylin–Eosin (H&E) Staining

Samples were harvested and fixed in 10% neutral formalin, dehydrated in a graded series of ethanol, and embedded in paraffin for routine H&E staining. All slides were examined by a pathologist with no prior knowledge of the treatment. The histological scores for epidermal and dermal regeneration used in this study were evaluated as previously described [19].

### 2.5. Masson's Trichrome Staining

Specimens were fixed in formalin and embedded in paraffin wax using routine laboratory techniques. Serial 5 *μ*m sections were cut and stained with Masson's trichrome to detect collagen fibers in the wounds. Deparaffinized sections were incubated with hematoxylin for 10 min, 0.5% hydrochloric acid/alcohol for 3 s, and 0.6% ammonia for 30 s. After washing in tap water for 1 min, sections were stained with Ponceau SP liquid for 1 h and then washed in tap water for 1 min. After 5 min in phosphomolybdic acid solution, sections were stained in water-soluble aniline blue for 5 min and then incubated in 1% acetic acid for 1 min. After routine alcohol- and xylene-based dehydration, the sections were fixed in 10% neutral buffered formalin for light microscopy. A pathologist who was blinded to the research design examined all sections and described any pathological changes.

Additionally, Image-Pro Plus (ver. 6.0) was used to scan and quantify the collagen deposition areas. The ratio of the fibrotic areas to the whole area was calculated as a relative objective index to assess the level of collagen fibers [20]. The results are presented as the mean of 10 different fields in each section.

### 2.6. Toluidine Blue Staining

Toluidine blue staining was used to assess the presence of MCs. Paraffin wax sections were deparaffinized and hydrated. Sections were stained with 1% toluidine blue for 10 min. The sections were washed under running water for 2 min and then differentiated with 95% and 100% alcohol. The sections were warmed and cleared in xylene and mounted with neutral resin. The number of MCs in the wound area (between the bilateral edges of the wound, 10 fields for each sample) was estimated by blinded pathologists.

### 2.7. Immunohistochemistry

Paraffin wax sections (5 *μ*m) were deparaffinized, washed three times in PBS for 5 min, and blocked with 5% serum for 30 min. The slides were subsequently incubated with primary antibodies against EGF (1 : 100), IL-1*β* (1 : 200), NG2 proteoglycan (1 : 100), desmin (1 : 200), or MMP-9(1 : 200) at 4°C overnight. After rinsing three times with PBS, the slides were incubated with horseradish peroxidase-labelled secondary antibodies at 37°C for 20–30 min and developed with 3,3′-diaminobenzidine tetrahydrochloride solution.

### 2.8. Western Blots

Skin tissue was homogenized in 500 *μ*L of cell lysate and transferred to a 1.5 mL microcentrifuge tube. The samples were lysed for 30 min and then centrifuged (14,000 ×g, 10 min, 4°C), and the supernatant was collected. Protein concentrations were determined using the BSA method, and the skin lysates were denatured at 95°C for 5 min in sample buffer. Then, 50 *μ*g of total protein was resolved by 10% SDS-PAGE and transferred to PVDF membranes. Membranes were blocked with 5% skim milk for 1 h at room temperature and incubated with primary antibodies against EGF (1 : 2000), NE (1 : 1500), IL-1*β* (1 : 1000), desmin (1 : 800), NG2 proteoglycan (1 : 2000), or MMP-9 (1 : 1000) overnight at 4°C. The membranes were then washed and incubated for 1 h at room temperature with an anti-rabbit or anti-mouse secondary antibody (1 : 10,000). After washing, the membranes were incubated with detection reagent (Immobilon Western Chemiluminescent HRP Substrate, Millipore Corp., Billerica, MA, USA). Band intensities were quantified using Image-Pro Plus (ver. 6.0). Protein levels were calculated relative to the sample's GAPDH level.

### 2.9. Statistical Analyses

The data of the size of wound belongs to the repeated measurement data, so we used the nonequidistant repeated measure variance analysis with the SPSS software (ver. 21; IBM). For the data of HE staining and Western blot (which belongs to the quantitative data), we found that the data in every group was homogeneity of variance after homogeneity test of variance. Then, we analyzed the data with independent-samples *t*-test. *p* values < 0.05 were considered to indicate statistical significance.

## 3. Results

### 3.1. The Glucose Levels in Both Groups

The glucose levels in the control group were not different from those in the experimental group on days 2 (*p* = 0.700), 5 (*p* = 0.309), 7 (*p* = 0.737), 10 (*p* = 0.322), 14 (*p* = 0.493), 17 (*p* = 0.333), and 21 (*p* = 0.743) (Table 1).

### 3.2. Wound Areas in the Diabetic Mice

**Figure 1:**
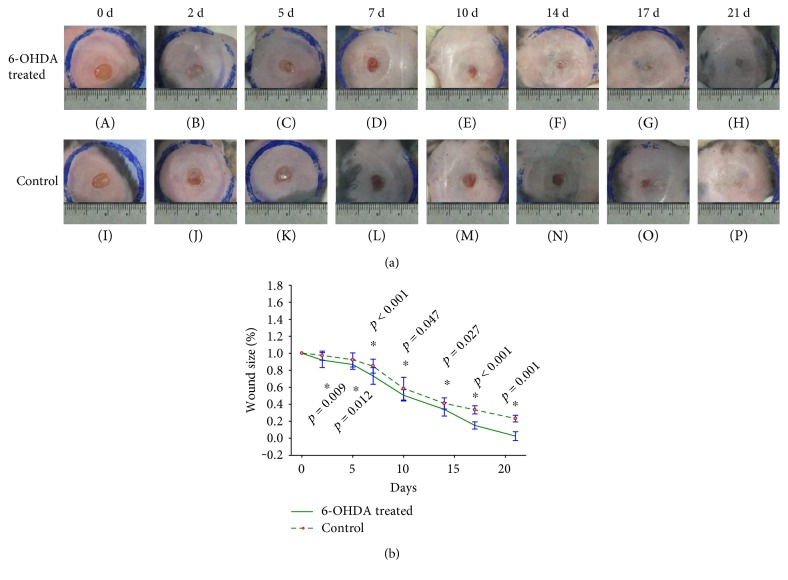
Wound size in 6-OHDA-treated and control groups after the establishment of full-thickness wounds in diabetic mice. (a) Wound size at different time points in the 6-OHDA-treated group (A–H) and the control group (I–P). (b) Significant differences in wound size in the 6-OHDA-treated group and the control group after the establishment of full-thickness wounds in diabetic mice. ^∗^*p* < 0.05, 6-OHDA-treated versus control.

All mice survived until they were sacrificed. Wound areas in the control group were larger than those in the experimental group on days 2 (*p* = 0.009), 5 (*p* = 0.012), 7 (*p* < 0.001), 10 (*p* = 0.047), 14 (*p* = 0.027), 17 (*p* < 0.001), and 21 (*p* = 0.001) (Figures 1(a) and 1(b)).

### 3.3. Histological Observation of Wounds through H&E Staining

**Figure 2:**
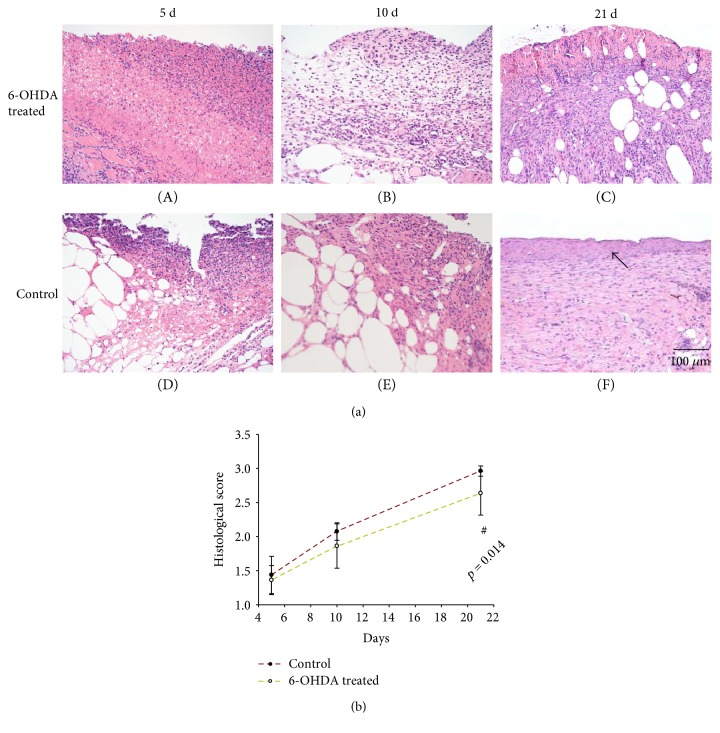
(a) H&E staining: the arrow indicates wound reepithelialization in the treatment and control groups (the bar corresponds to 100 *μ*m). (b) The histological scores of epidermal and dermal regeneration in the 6-OHDA-treated and control groups. ^#^*p* < 0.05, 6-OHDA-treated versus control.

Skin structure in diabetic mice was shown in Figure S1 available online at https://doi.org/10.1155/2017/7614685, which had continuous epidermal structure and hair follicles. Many inflammatory cells were observed in the wounds during the early stage of healing. Throughout the entire wound-healing process, collagen fibers increased steadily, and the wound surface was gradually covered by epidermal cells (Figure 2(a)). The histological scores for epidermal and dermal regeneration in the 6-OHDA-treated group were lower than those in the control group on days 5 (*p* = 0.550), 10 (*p* = 0.107), and 21 (*p* = 0.014) (Figure 2(b)).

### 3.4. Histological Observation of Wounds through Masson's Trichrome Staining

**Figure 3:**
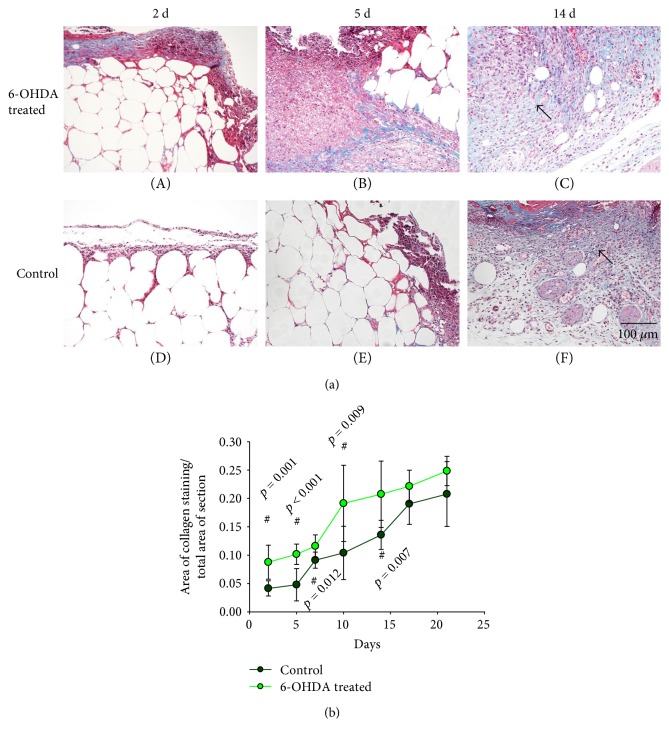
(a) Histological analyses of Masson staining in dorsal skin after full-thickness wounds at different time points in the 6-OHDA-treated group (A–C) and the control group (D–F). The bar corresponds to 100 *μ*m. (b) The ratio of fibrotic area to the whole area in diabetic wounds. ^#^*p* < 0.05, 6-OHDA-treated versus control.

There are many blue-stained collagen fibers in the extracellular matrix of the skin in diabetic mice (Figure S2). On day 2, the skin wounds showed obvious inflammatory responses. During the healing process, epithelial cells proliferated and migrated to the wound bed, and the number of collagen fibers increased in the wounds and wound edges (Figure 3(a)). The ratio of the fibrotic area to the whole area was higher in the treatment group than in the control group on days 2 (*p* = 0.001), 5 (*p* < 0.001), 7 (*p* = 0.012), 10 (*p* = 0.009), 14 (*p* = 0.007), 17 (*p* = 0.076), and 21 (*p* = 0.087) (Figure 3(b)).

### 3.5. MC Staining

**Figure 4:**
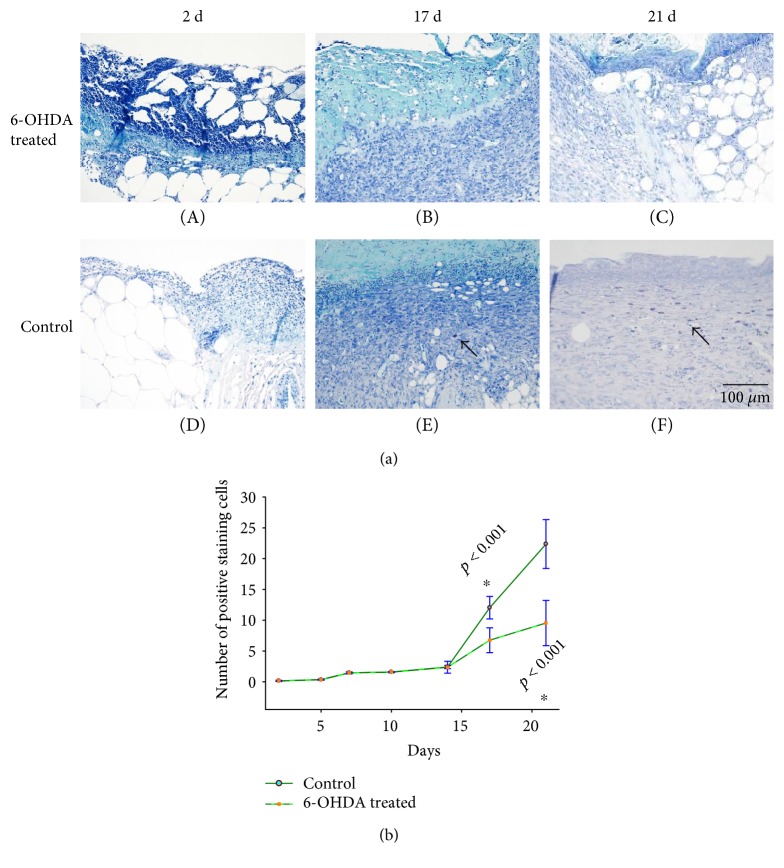
(a) Histological analyses of dorsal skin stained with toluidine blue after full-thickness wounds at different time points in the 6-OHDA-treated group (A–C) and the control group (D–F). Arrows indicate positive staining within MCs. The bar corresponds to 100 *μ*m. (b) The number of MCs in each field in the 6-OHDA-treated and control groups. ^∗^*p* < 0.05, 6-OHDA-treated versus control.

There were some purple cells (MCs) in the skin of diabetic mice without wound (Figure S3). Few MCs were observed in the skin wounds by toluidine blue staining on days 2, 5, 7, or 10. MCs were localized primarily in healthy skin tissue and the wound edges. MCs became more apparent during the healing of the diabetic wounds (Figure 4(a)). Fewer MCs were observed in each field in the treatment group than in the control group on days 2 (*p* = 0.642), 5 (*p* = 0.513), 7 (*p* = 1.000), 10 (*p* = 0.549), 14 (*p* = 0.917), 17 (*p* < 0.001), and 21 (*p* < 0.001) (Figure 4(b)).

### 3.6. Expression of Proteins in Wounds

**Figure 5:**
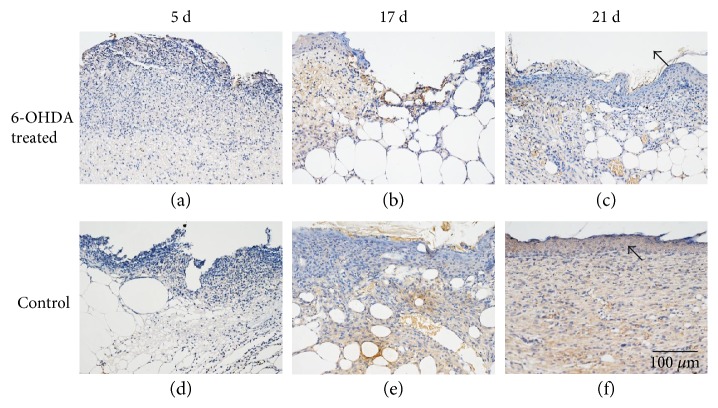
EGF immunohistochemistry. EGF is expressed in normal epithelial cells and fibroblasts. Arrows indicate positive staining within epithelial cells. The bar corresponds to 100 *μ*m.

**Figure 6:**
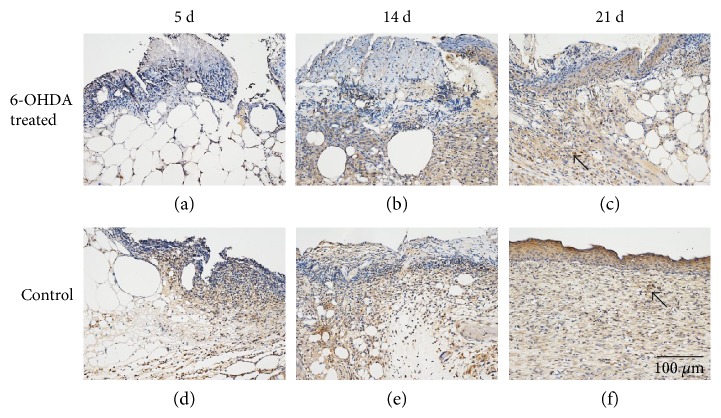
IL-1*β* immunohistochemistry. IL-1*β* is widely expressed on fibroblasts, macrophages, and epidermal cells. Arrows indicate positive staining within fibroblasts. The bar corresponds to 100 *μ*m.

**Figure 7:**
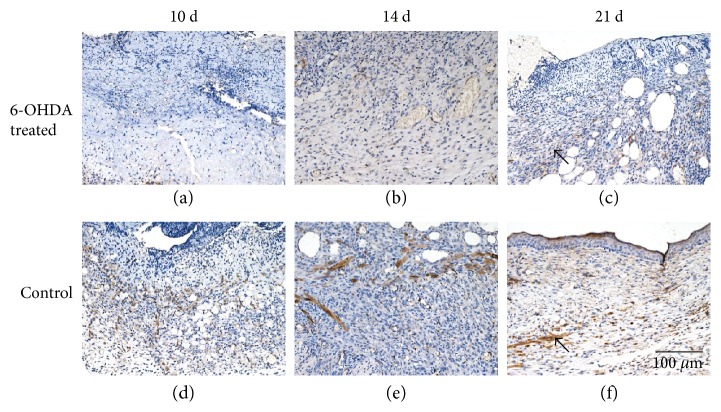
Immunohistochemical staining of desmin in the wounds of diabetic mice. Desmin is expressed by pericytes. The bar corresponds to 100 *μ*m.

**Figure 8:**
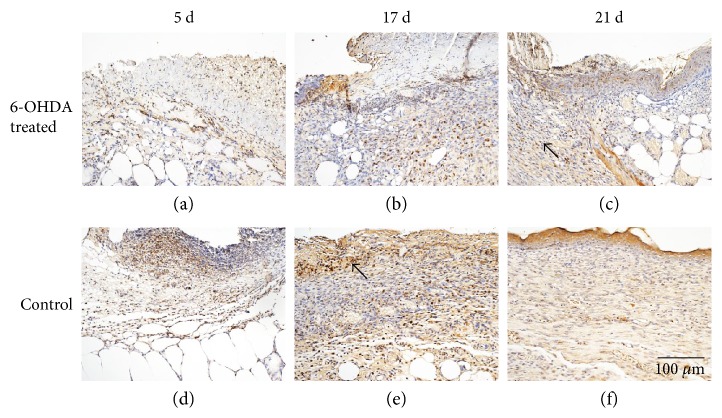
Immunohistochemical staining of NG2 proteoglycan in the wounds of diabetic mice. NG2 proteoglycan is expressed by the mural cell component of mouse neovascular structures. The bar corresponds to 100 *μ*m.

**Figure 9:**
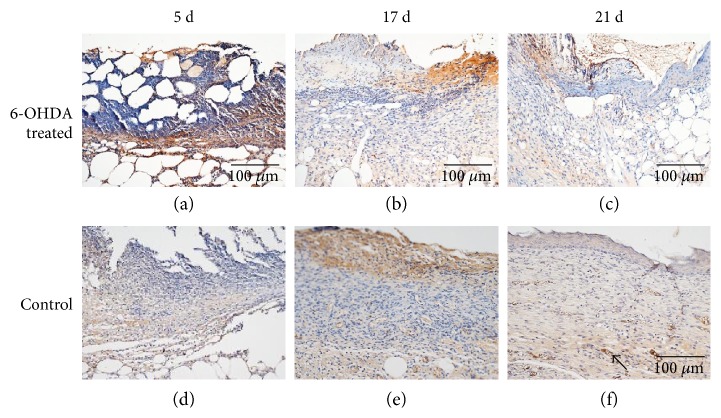
Immunohistochemical staining of MMP-9 in the wounds of diabetic mice. MMP-9 is widely distributed in various tissues and bodily fluids. The bar corresponds to 100 *μ*m.

The expressions of EGF, IL-1*β*, desmin, NG2 proteoglycan, and MMP-9 in the skin of diabetic mice are indicated in Figures 4, 5, 6, 7, and 8. Immunohistochemistry results for EGF, IL-1*β*, desmin, NG2 proteoglycan, and MMP-9 are shown in Figures 5, 6, 7, 8, and 9. EGF was found in normal epithelial cells (Figure 5). IL-1*β* is a member of the IL-1 cytokine family and is produced by activated macrophages as a proprotein to induce various acute-phase reactions [21] (Figure 6). Desmin filaments exist in smooth muscle cells [22] (Figure 7). NG2 proteoglycan was invariably expressed by the mural cell component of mouse neovascular structures [23] (Figure 8). MMP-9 (Figure 9) is a matrix metalloproteinase and was widely distributed in various tissues and body fluids.

**Figure 10:**
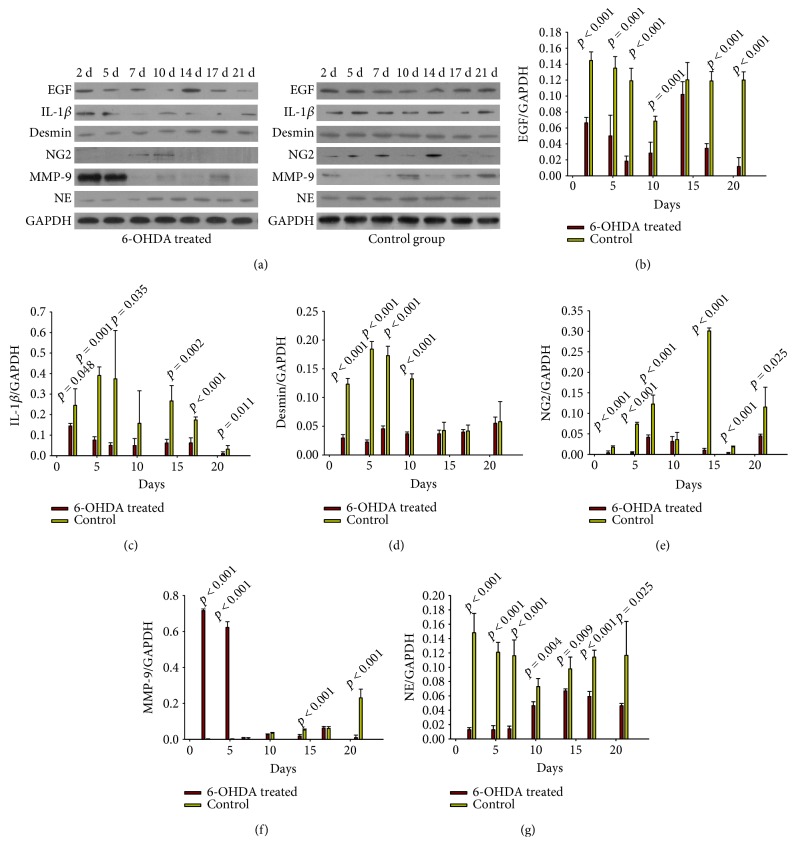
(a) Western blot results. (b)–(g) Relative protein expression levels of EGF, IL-1*β*, desmin, NG2, MMP-9, and NE in the wounds of diabetic mice. Protein expression levels were calculated relative to GAPDH from the same sample. The values shown are the mean ± standard deviations, *n* = 4 specimens per group at each time point. *p* < 0.05, 6-OHDA treated versus control (one-way analysis of variance (ANOVA)).

Protein levels detected by Western blot are shown in Figure 10(a). EGF was reduced in the treatment group on days 2 (*p* < 0.001), 5 (*p* = 0.001), 7 (*p* < 0.001), 10 (*p* = 0.001), 14 (*p* = 0.224), 17 (*p* < 0.001), and 21 (*p* < 0.001) (Figure 10(b)). The levels of IL-1*β* in the treatment group were lower on days 2 (*p* = 0.048), 5 (*p* = 0.001), 7 (*p* = 0.139), 10 (*p* = 0.408), 14 (*p* = 0.008), 17 (*p* < 0.001), and 21 (*p* = 0.116) after injury (Figure 10(c)) compared with those in the control group. Desmin expression in the treatment group was significantly less than that in the control group on days 2 (*p* < 0.001), 5 (*p* < 0.001), 7 (*p* < 0.001), 10 (*p* < 0.001), 14 (*p* = 0.442), 17 (*p* = 0.698), and 21 (*p* = 0.869) (Figure 10(d)). The expression of NG2 proteoglycan in the 6-OHDA-treated group was lower than that in the control group on days 2 (*p* < 0.001), 5 (*p* < 0.001), 7 (*p* < 0.001), 10 (*p* = 0.001), 14 (*p* < 0.001), 17 (*p* < 0.001), and 21 (*p* < 0.001) (Figure 10(e)). MMP-9 expression in the experimental group exceeded than that of the control group on days 2 (*p* < 0.001), 5 (*p* < 0.001), and 7 (*p* = 0.115). However, MMP-9 expression in the control group increased and surpassed that of the treatment group on days10 (*p* = 0.056), 14 (*p* < 0.001), 17 (*p* = 0.777), and 21 (*p* < 0.001) (Figure 10(f)). NE levels decreased significantly after sympathectomy on days 2 (*p* < 0.001), 5 (*p* < 0.001), 7 (*p* < 0.001), 10 (*p* = 0.004), 14 (*p* = 0.009), 17 (*p* < 0.001), and 21 (*p* = 0.025) (Figure 10(g)).

## 4. Discussion

Wound healing requires successive phases of inflammation, cell proliferation, cell migration, angiogenesis, and reepithelialization [5]. Sympathetic denervation, prolonged inflammatory responses, and impaired angiogenesis are often present in patients with DFUs. The relationship between these three features in the diabetic foot is not completely clear. Sympathetic nerve activity may correlate with reepithelialization, neurogenic inflammation [12], collagen metabolism, and angiogenesis [6]. The role of sympathetic nerve failure in DFUs remains controversial. Sympathetic nerves are located near pericytes in the microvessels [6]. It has not been reported how sympathetic denervation affects inflammation and angiogenesis in DFUs.

6-OHDA can reduce NE concentrations [24] and can be used to create animal models of sympathetic denervation, which was also shown in our study (Figure 0()). We show here that chemical sympathectomy with 6-OHDA accelerates wound contraction in diabetic wounds (Figures 1(a) and 1(b)). There were more collagen fibers in the 6-OHDA-treated group than in the control group (Figures 3(a) and 3(b)). This result differs from the report of Kokobelian et al. [10], who concluded that sympathectomy was ineffective for the treatment of DFUs.

Epithelialization is an important aspect of the healing process. We found that the reepithelialization rate in wounds was reduced after sympathectomy (Figures 2(a) and 2(b)), suggesting that the delayed reepithelialization in DFUs [25] may be due to sympathetic nerve failure. Previous studies also showed that chemical sympathectomy with 6-OHDA can delay reepithelialization in cutaneous wounds [12, 14]. EGF plays an essential role in wound healing by stimulating epidermal and dermal regeneration [26]. EGF was found to be decreased in diabetic wounds after sympathectomy (Figures 5 and 10). Thus, 6-OHDA may delay reepithelialization in diabetic wounds by reducing their EGF levels.

MCs participate in the inflammatory process of wound healing and in obesity and diabetes [27]. The number of degranulated MCs was found to be increased in the unwounded forearm and foot skin of diabetic patients and in the unwounded dorsal skin of diabetic mice [28]. MCs recruit inflammatory cells directly and indirectly [27] and act as transducers between peripheral nerves and local inflammatory events [29]. We found few MCs in inflamed skin wounds (Figure 4(a)), but MCs began to increase rapidly during the remodeling phase. This finding is consistent with a report by Nishikori et al. [30]. Additionally, in our experiment, MCs were significantly decreased after sympathectomy (Figure 4(b)). Similarly, Souza et al. [14] demonstrated that chemical sympathectomy led to reduced MC migration during cutaneous wound healing in rats. This finding suggests that a reduction in MCs may promote diabetic wound healing in 6-OHDA-treated animals.

IL-1*β* is upregulated during the inflammatory phase of diabetic wounds [31]. Inhibiting the IL-1*β* pathway in the wounds of diabetic mice led to an increase in the levels of wound growth factors and improved wound healing [32]. We found that 6-OHDA reduced the levels of IL-1*β* in diabetic wounds (Figures 6 and 10). Kim et al. [12] showed that 6-OHDA significantly reduced neurogenic inflammation in animal skin incisions. This finding suggests that sympathetic denervation can inhibit the prolonged inflammatory response induced by IL-1*β* and MCs in diabetic wounds.

The process of angiogenesis includes endothelial cell activation, the degradation of the vascular basement membrane, and vascular sprouting [33]. Angiogenesis depends upon the balance of pericytes and endothelial cells. Pericytes embedded within the basement membrane of capillaries and postcapillary venules play an important role in endothelial cell proliferation, migration, and stabilization [34]. An imbalance of pericytes and endothelial cells will impair the development of functional capillaries in DFUs [35]. Few studies have evaluated the effect of sympathetic denervation on angiogenesis in DFUs.

In this experiment, lower expression levels of desmin and NG2 proteoglycan were observed in the 6-OHDA-treated group (Figures 7, 8, and 10). Previous studies demonstrated that surgical sympathectomy may cause a reduction in pericyte markers (platelet-derived growth factor-BB (PDGF-BB) and NG2 proteoglycan) in rat retinas [36]. Thus, pericyte proliferation in diabetic wounds may be inhibited by sympathectomy. Pericytes are derived from hematopoietic stem cells [37]. The number of hematopoietic stem and progenitor cells (HSPC) mobilized by granulocyte colony-stimulating factor was dramatically reduced in 6-OHDA-lesioned mice [38]. Adrenergic receptors were found to be present in endothelial cells and pericytes [6]. Therefore, reduced levels of pericytes in 6-OHDA-treated diabetic mice may be due to abrogated HSPC migration.

Pericytes are crucial for the survival of endothelial cells and may control endothelial cell proliferation [39]. Pericyte-derived NG2 promotes endothelial cell migration and morphogenesis during the early stages of neovascularization [40]. The number of endothelial cells and pericytes was significantly reduced in the retina of NG2-knockout mice [41]. This finding suggests that a reduction in pericytes after sympathectomy may lead to a decrease in endothelial cells.

MMP-9 is involved in the dynamic remodeling of the ECM, which is essential for all stages of angiogenesis [33]. An increased ratio of serum MMP-9 has been observed in DFU patients [42]. In our study, MMP-9 expression in the treatment group was significantly higher than in the control group on days 2 and 5. With the effect of sympathetic denervation gradually diminishing, MMP-9 expression increased in the control group and surpassed the treatment group on days10, 14, 17, and 21. Thus, increased MMP-9 expression in diabetic wounds may result in part from sympathetic denervation.

Previous studies found that inflammatory factors, such as MCs and IL-1*β*, were upregulated in the skin and wounds of diabetic patients. Excessive inflammatory responses are harmful in diabetic wounds; thus, the control of inflammation by 6-OHDA can promote wound contraction. The level of angiogenesis in wounds often correlates with the inflammatory response, largely because inflammatory cells produce an abundance of proangiogenic mediators [43]. In this study, the selective reduction of inflammation and angiogenesis were both found after sympathectomy in diabetic wounds. The relationship between inflammation and angiogenesis is complex, and further research is required to determine which process plays the more predominant role in diabetic wound healing.

In conclusion, as Souza et al. 's report in normal rats [14], sympathetic denervation accelerates wound contraction but delays reepithelialization in diabetic mice. We further proved that 6-OHDA decreased EGF, IL-1*β* levels, and the number of mast cells (MCs). Besides, the sympathetic denervation caused by 6-OHDA led to reduced pericyte proliferation in diabetic wounds, which might explain the vascular dysfunction after sympathetic nerve loss in diabetic wounds.

## Supplementary Material

Figure S1. H&E-stained histology in the skin of diabetic (db/db) mice. Figure S2. Collagen fibers in the skin of diabetic (db/db) mice by Masson trichrome staining. Figure S3.Mast cells in the skin of diabetic (db/db) mice by Toluidine blue staining. Figure S4. EGF expression in the skin of diabetic (db/db) mice by immunohistochemistry. Figure S5. IL-1β expression in the skin of diabetic (db/db) mice by immunohistochemistry. Figure S6. Desmin expression in the skin of diabetic (db/db) mice by immunohistochemistry. Figure S7. NG2 expression in the skin of diabetic(db/db) mice by immunohistochemistry. Figure S8. MMP-9 expression in the skin of diabetic(db/db) mice by immunohistochemistry.

## Figures and Tables

**Table 1:** The glucose levels in both 6-OHDA-treated and control groups during this experimental phase.

	Days						
	2 d	5 d	7 d	10 d	14 d	17 d	21 d
Groups							
Control	19.95 ± 0.74	20.83 ± 1.86	20.78 ± 3.72	20.30 ± 0.66	21.86 ± 3.96	21.95 ± 3.10	21.90 ± 3.73
6-OHDA treated	20.15 ± 0.66	19.65 ± 1.01	20.08 ± 1.40	21.90 ± 2.89	20.36 ± 0.81	20.15 ± 0.66	21.10 ± 2.80
*p* value	0.700	0.309	0.737	0.322	0.493	0.333	0.743
