# Supplementary material for: Sympathetic Denervation Accelerates Wound Contraction but Inhibits Reepithelialization and Pericyte Proliferation in Diabetic Mice
Source: J Diabetes Res. 2017 Sep 24;2017:7614685. doi: 10.1155/2017/7614685 (PMC5632918; doi:10.1155/2017/7614685)
Supplement: Supplementary file 1 — Figure S1. H&E-stained histology in the skin of diabetic (db/db) mice. Figure S2. Collagen fibers in the skin of diabetic (db/db) mice by Masson trichrome staining. Figure S3.Mast cells in the skin of diabetic (db/db) mice by Toluidine blue staining. Figure S4. EGF expression in the skin of diabetic (db/db) mice by immunohistochemistry. Figure S5. IL-1β expression in the skin of diabetic (db/db) mice by immunohistochemistry. Figure S6. Desmin expression in the skin of diabetic (db/db) mice by immunohistochemistry. Figure S7. NG2 expression in the skin of diabetic(db/db) mice by immunohistochemistry. Figure S8. MMP-9 expression in the skin of diabetic(db/db) mice by immunohistochemistry. [file 7614685.f1.doc]

Supplementary Data


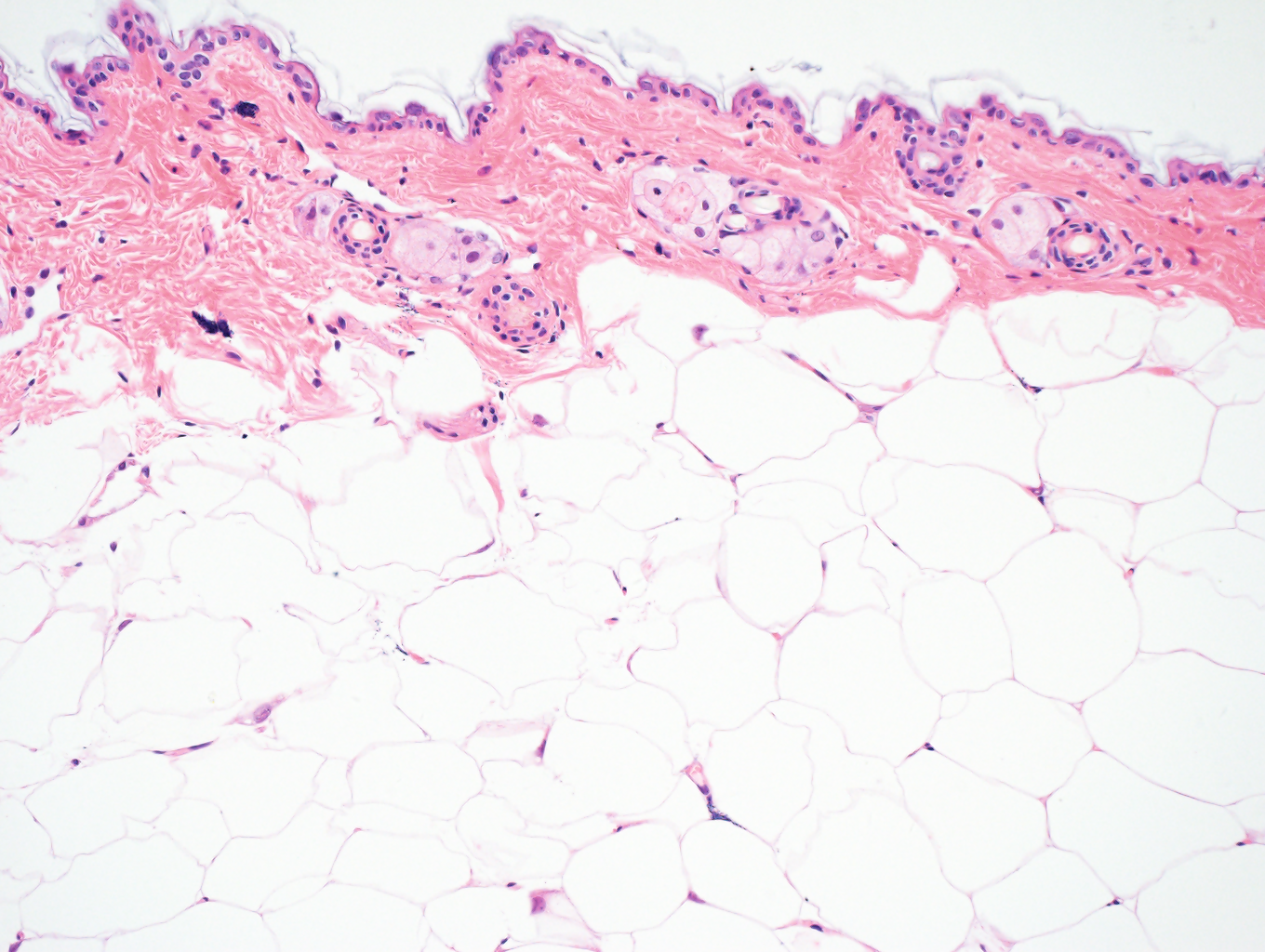


Fig.S1.H&E-stained histology in the skin of diabetic (db/db) mice


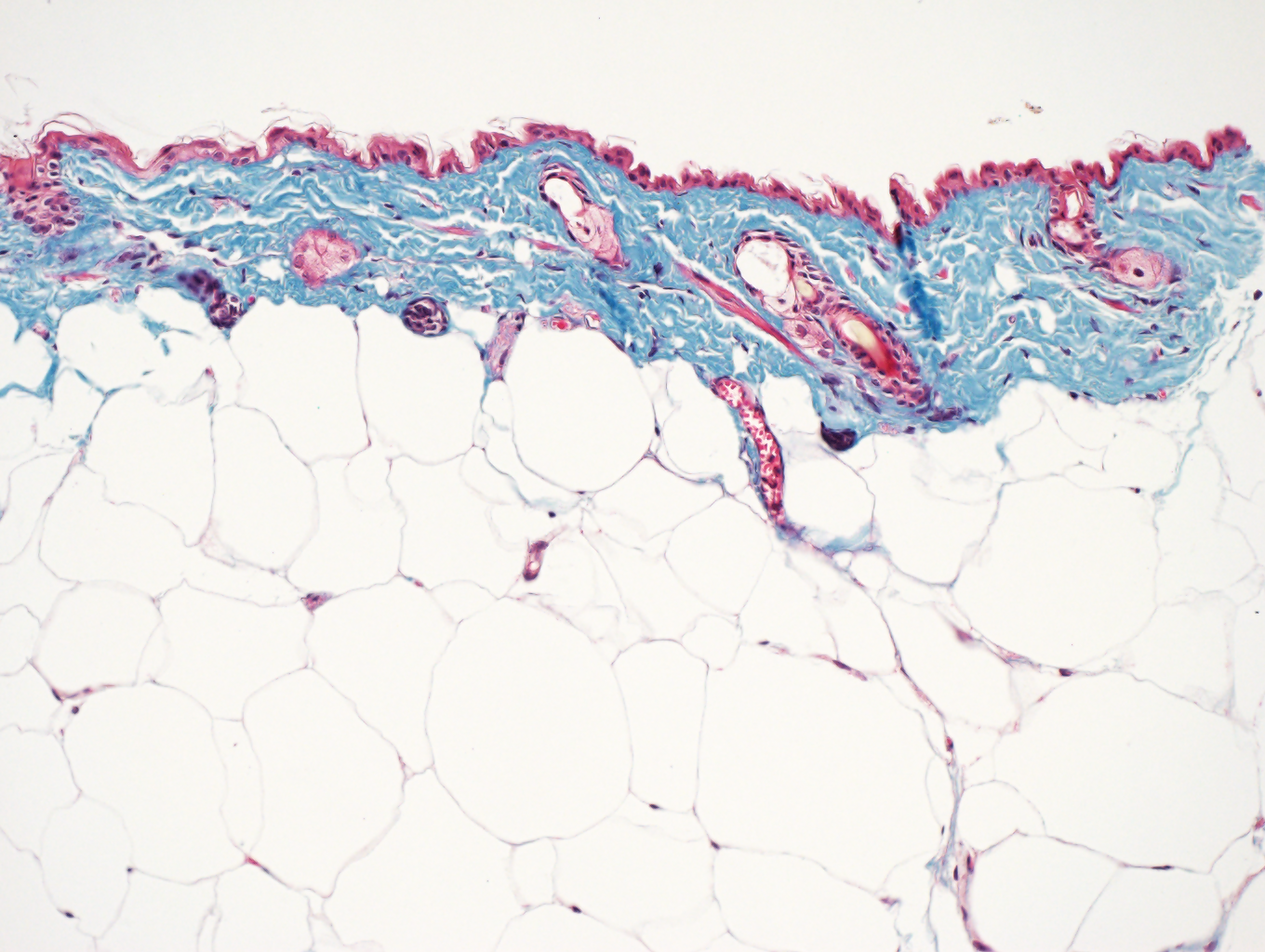


Fig.S2. Collagen fibers in the skin of diabetic (db/db) mice by [Masson trichrome staining](http://www.bioon.com/experiment/histolog4/59786.shtml)


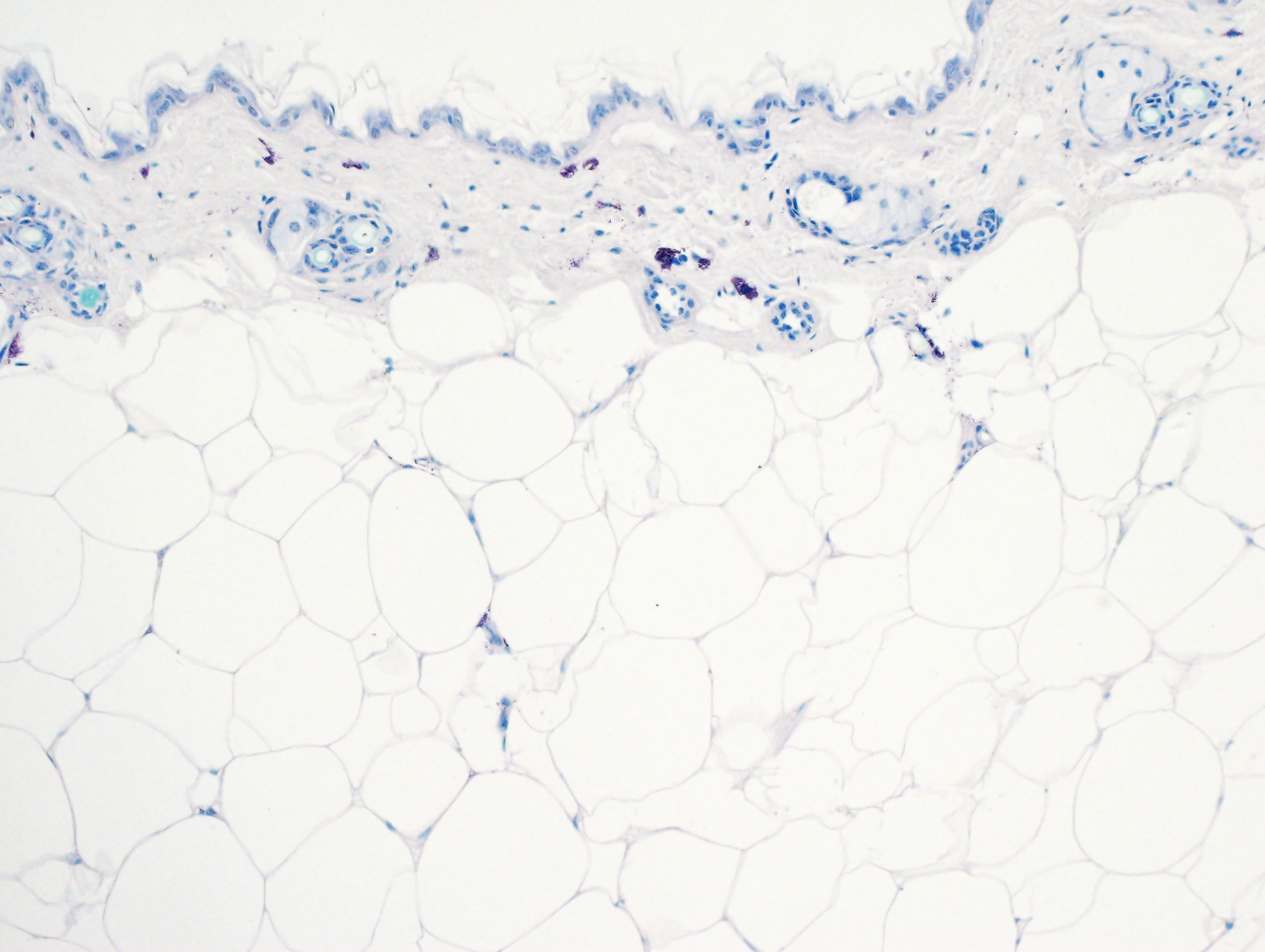


Fig.S3.Mast cells in the skin of diabetic (db/db) mice by Toluidine blue staining


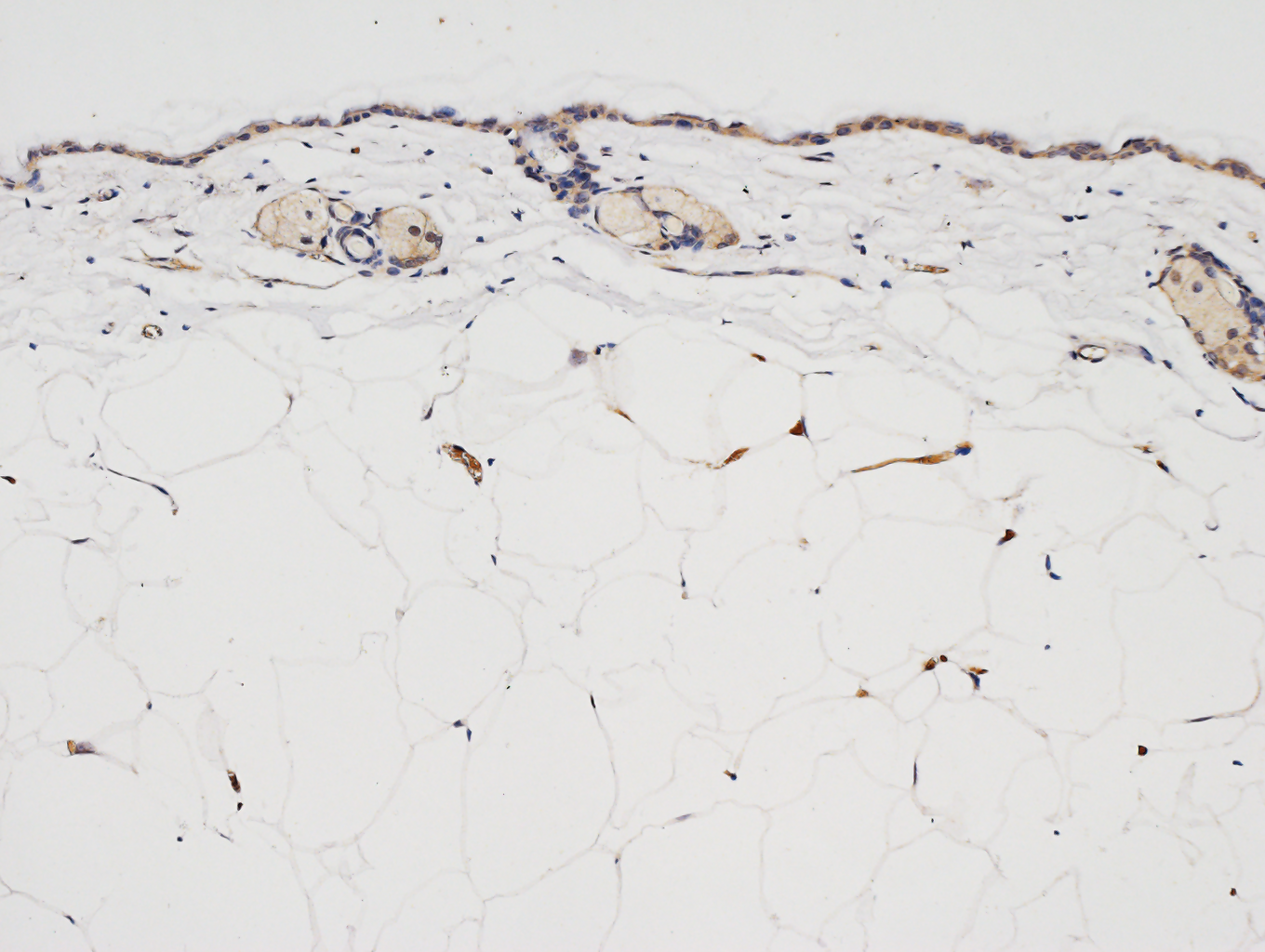


Fig.S4.EGF expression in the skin of diabetic (db/db) mice by immunohistochemistry


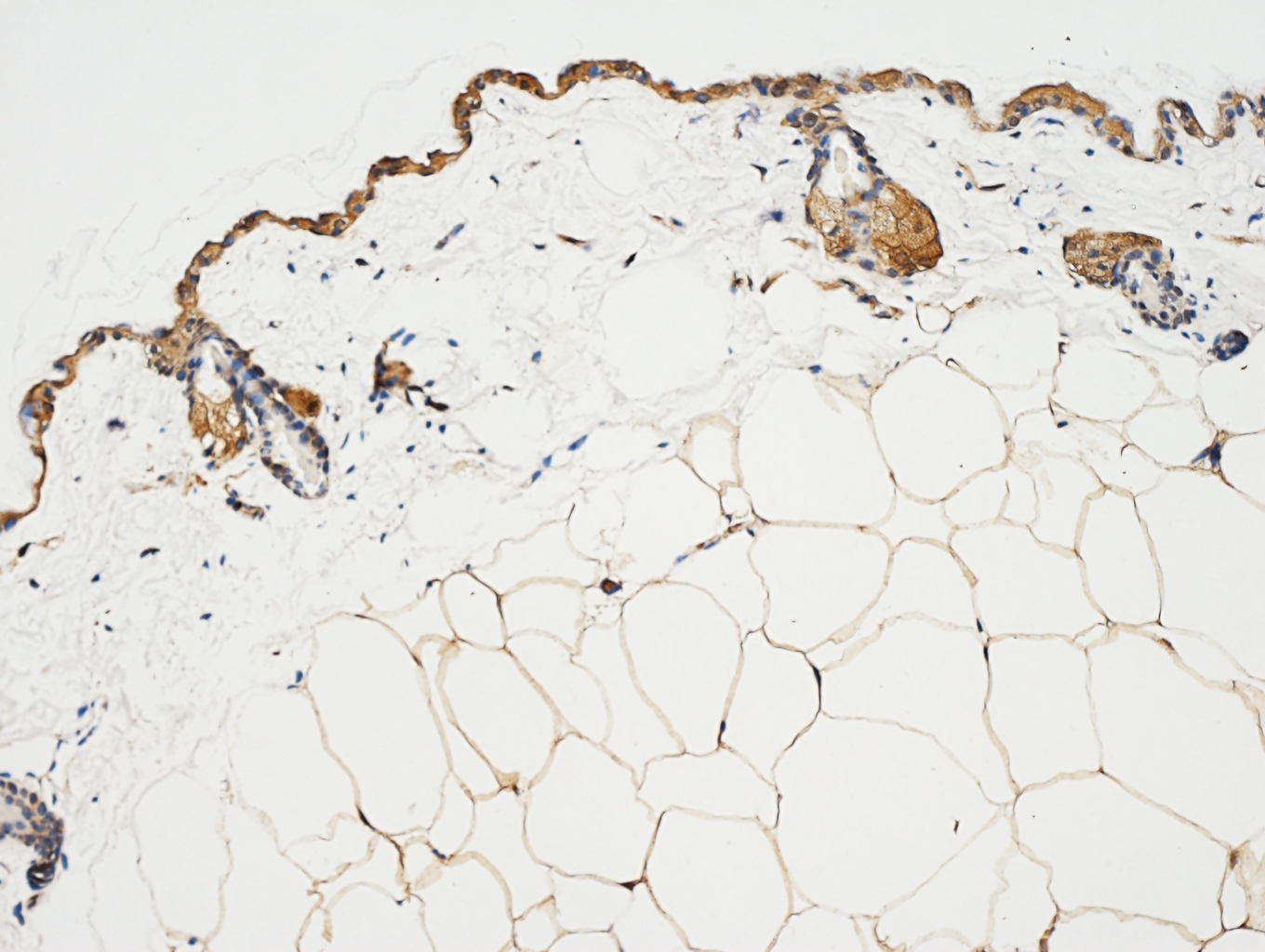


Fig.S5. IL-1β expression in the skin of diabetic (db/db) mice by immunohistochemistry


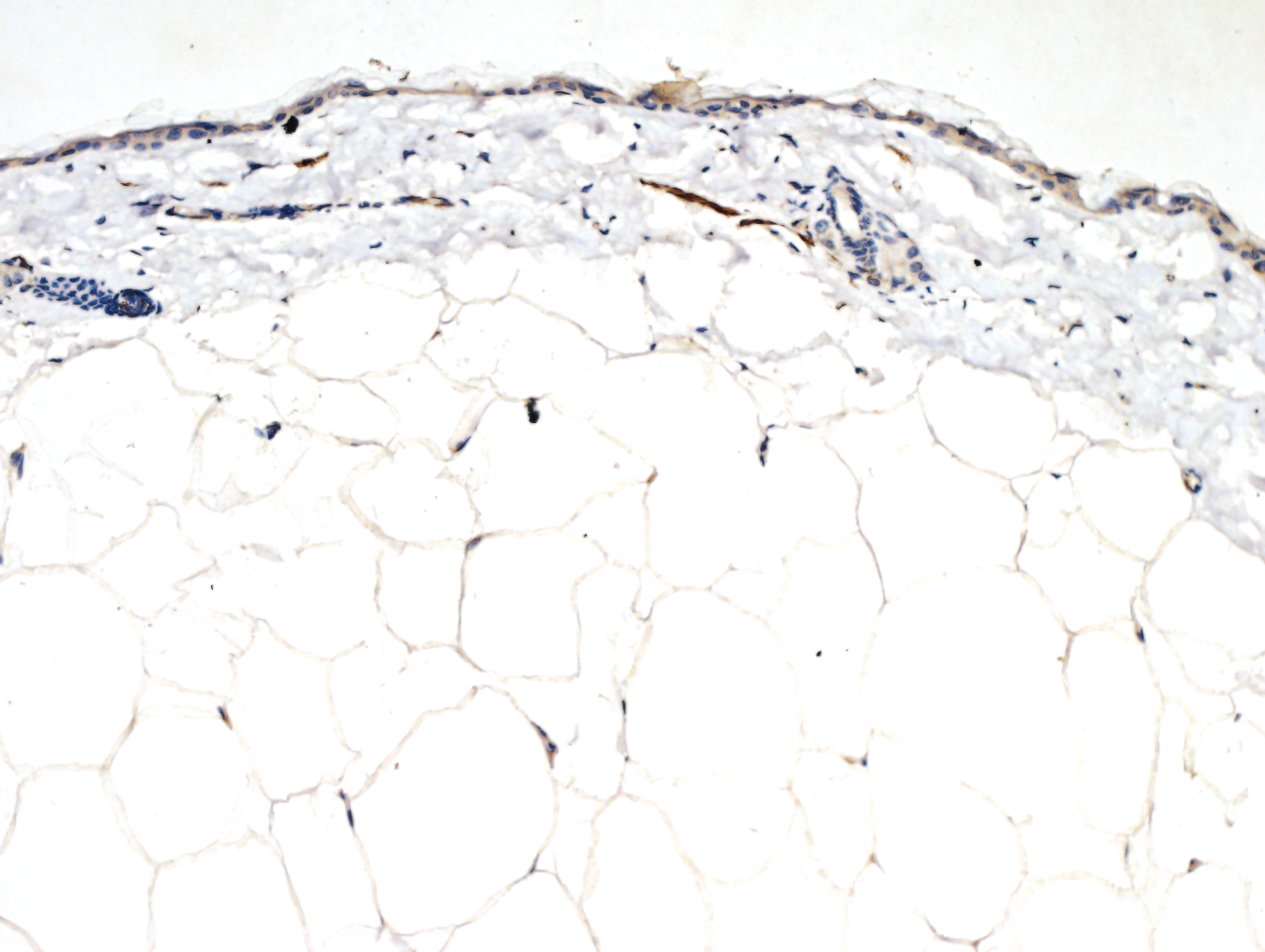


Fig.S6. Desmin expression in the skin of diabetic (db/db) mice by immunohistochemistry


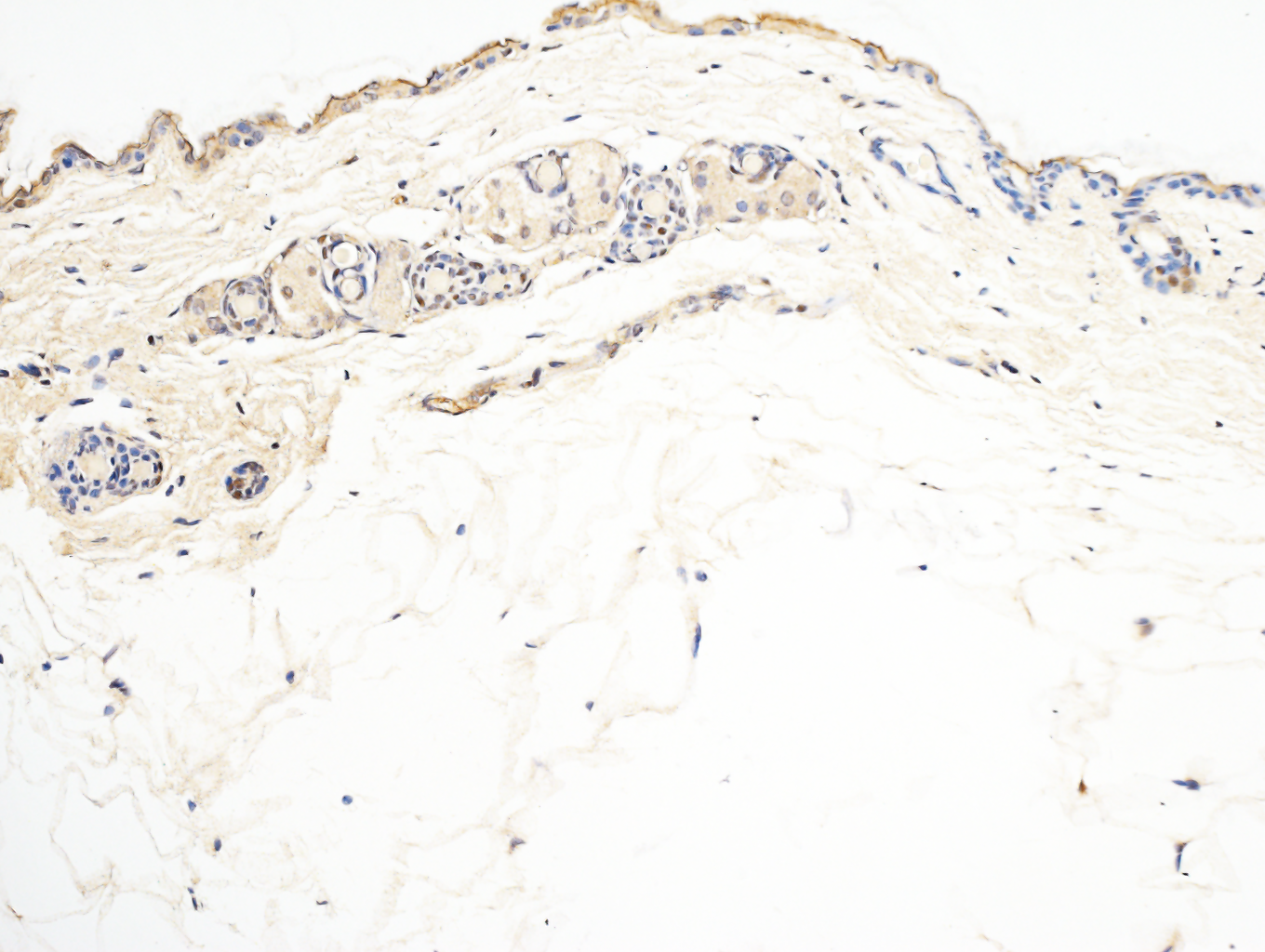


Fig.S7. NG2 expression in the skin of diabetic(db/db) mice by immunohistochemistry


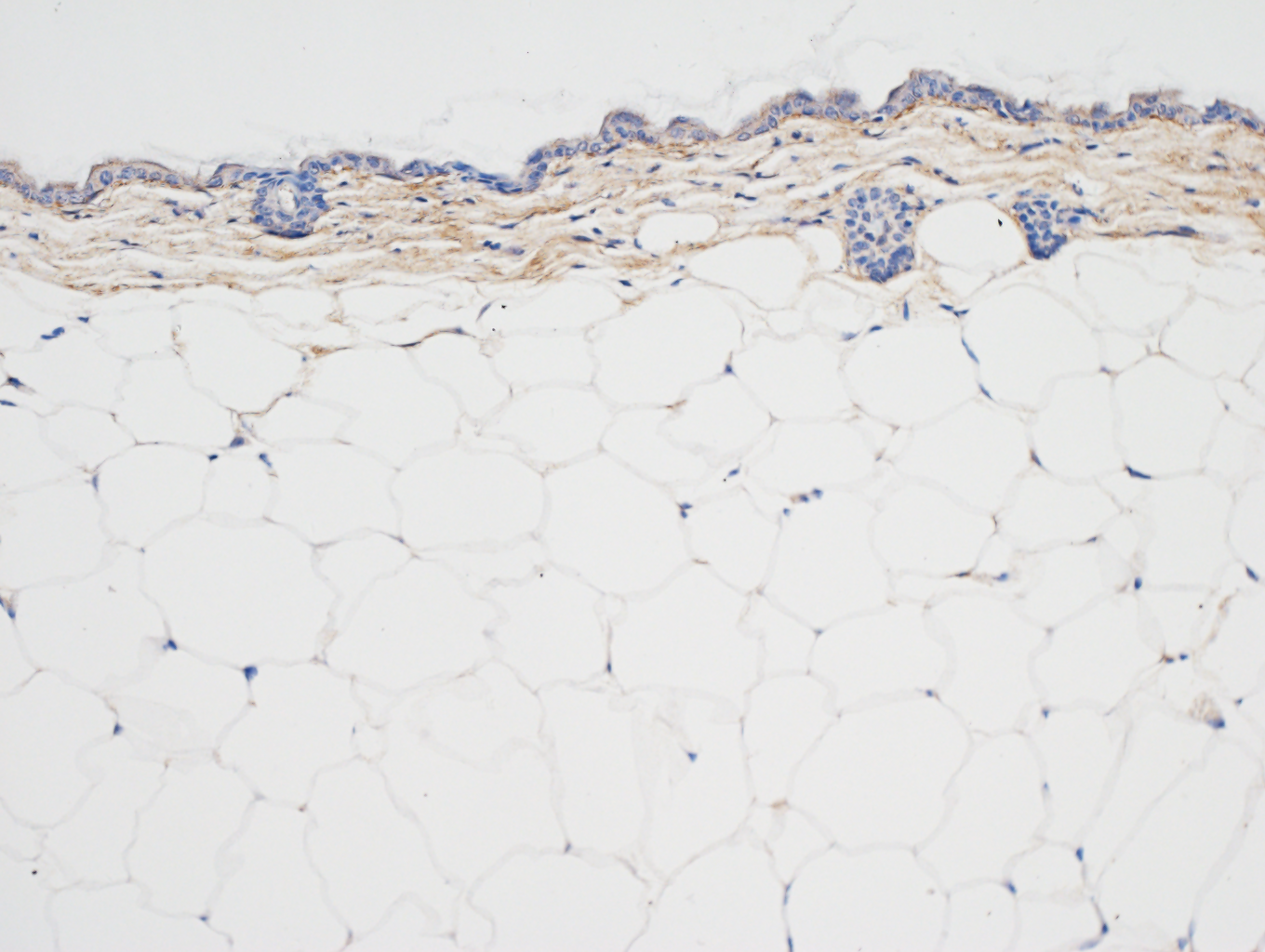


Fig.S8. MMP-9 expression in the skin of diabetic(db/db) mice by immunohistochemistry
